# Quantifiable Biomarkers of Normal Aging in the Japanese Medaka Fish (*Oryzias latipes*)

**DOI:** 10.1371/journal.pone.0013287

**Published:** 2010-10-11

**Authors:** Lingling Ding, Wendy W. Kuhne, David E. Hinton, Jian Song, William S. Dynan

**Affiliations:** 1 Institute of Molecular Medicine and Genetics, Medical College of Georgia, Augusta, Georgia, United States of America; 2 Department of Anatomy and Embryology, Wuhan University School of Medicine, Hubei, China; 3 Savannah River National Laboratory, Savannah River Site, Aiken, South Carolina, United States of America; 4 Nicholas School of the Environment, Duke University, Durham, North Carolina, United States of America; National Institute on Aging, United States of America

## Abstract

**Background:**

Small laboratory fish share many anatomical and histological characteristics with other vertebrates, yet can be maintained in large numbers at low cost for lifetime studies. Here we characterize biomarkers associated with normal aging in the Japanese medaka (*Oryzias latipes*), a species that has been widely used in toxicology studies and has potential utility as a model organism for experimental aging research.

**Principal Findings:**

The median lifespan of medaka was approximately 22 months under laboratory conditions. We performed quantitative histological analysis of tissues from age-grouped individuals representing young adults (6 months old), mature adults (16 months old), and adults that had survived beyond the median lifespan (24 months). Livers of 24-month old individuals showed extensive morphologic changes, including spongiosis hepatis, steatosis, ballooning degeneration, inflammation, and nuclear pyknosis. There were also phagolysosomes, vacuoles, and residual bodies in parenchymal cells and congestion of sinusoidal vessels. Livers of aged individuals were characterized by increases in lipofuscin deposits and in the number of TUNEL-positive apoptotic cells. Some of these degenerative characteristics were seen, to a lesser extent, in the livers of 16-month old individuals, but not in 6-month old individuals. The basal layer of the dermis showed an age-dependent decline in the number of dividing cells and an increase in senescence-associated β-galactosidase. The hearts of aged individuals were characterized by fibrosis and lipofuscin deposition. There was also a loss of pigmented cells from the retinal epithelium. By contrast, age-associated changes were not apparent in skeletal muscle, the ocular lens, or the brain.

**Significance:**

The results provide a set of markers that can be used to trace the process of normal tissue aging in medaka and to evaluate the effect of environmental stressors.

## Introduction

A number of invertebrate and vertebrate model organisms have been used to explore the root causes of aging. Fish are useful models for this purpose because most of their organ systems, including the brain, the heart, the hematopoietic system, and the digestive and excretory organs, are similar to those of other vertebrates. Different fish species show extraordinarily diverse patterns of aging. For example, the Pacific salmon undergoes rapid senescence and death at first spawning, whereas rockfish species exhibit indeterminate growth with very slow or negligible aging [1], [2]. Many small laboratory fish such as the guppy, the zebrafish, and the medaka, and the annual killifish are characterized by a regular time-dependent aging similar to that of other vertebrates [2], [3], [4], [5]. The rationale for use of these species in experimental gerontology includes their short lifespans, the relatively low cost of maintaining large numbers of individuals for lifetime studies, and in many cases, a well-developed genetics.

Fish accumulate many of the same age-associated biomarkers that have been characterized in other vertebrates. These include senescence associated β-galactosidase (SA β-gal), oxidized proteins, and lipofuscin (non-degradable pigment granules composed of peroxidized lipids [1], [4], [6]. These markers are thought to arise as a result of a dysfunctional mitochondrial-lysosomal axis in senescent tissue [7], [8], [9]. High levels of SA β-gal have been used as the basis of a zebrafish genetic screen, which identified homologues of genes that have been implicated in aging or genome maintenance in other organisms [10]. Together, these data underscore the similarities in the pathophysiology and genetics of aging between fish and other species.

The Japanese medaka (*Oryzias latipes*) is a well-characterized teleost model organism with potential utility in experimental aging research. It has a fully sequenced genome, and many genetic tools are available, including transgenesis and target-selected mutagenesis, which can be used to manipulate expression of aging-related genes (reviewed in [11], [12], [13], [14]). Lifespan has been characterized in medaka populations [15], [16], [17]. Telomeres and telomerase, which have been linked to aging in other systems, have also been characterized in the medaka [18], [19], [20]. One of the attractive aspects of medaka is that they have been widely used in toxicology research [21], [22], [23]. In particular, their ability to tolerate a range of environmental conditions has made them useful for investigating the effects of exposure to environmental stressors outside the conventional biological laboratory setting [24], [25], [26], [27]. These characteristics suggest that medaka might be a useful model to investigating the interaction between genotoxic agents and aging. Indeed, there is one early report suggesting that a single exposure to ionizing radiation during development significantly shortens lifespan [16].

Here we investigate a number of tissues that have been shown to undergo age-related changes in other models. These include: liver, skin, skeletal muscle, heart, eye, and brain. Results showed extensive morphologic changes in the liver, accompanied by quantifiable changes in the number and size of lipofuscin deposits and TUNEL-positive apoptotic cells. Age-associated changes were also noted in the skin, heart and retina. Age-associated changes in other tissues, including the ocular lens, skeletal muscle, and the central nervous system, were not evident. These data, which have previously been lacking, will expand the utility of medaka for aging research.

## Results

### Overall growth and survival

**Figure 1:**
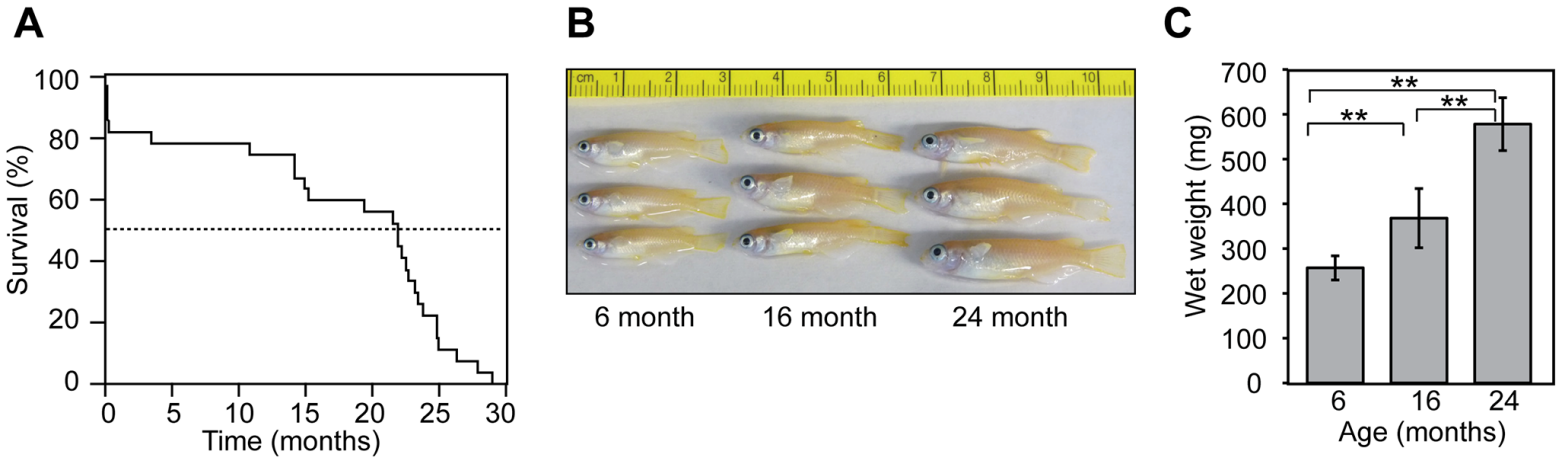
Survival and growth of medaka under laboratory conditions. (**A**) Kaplan-Meier survival analysis. Time post-hatching is shown in months. Dashed line indicates 50% survival. (**B**) Gross appearance of representative 6, 16, and 24-month-old male medaka. (**C**) Wet weight of medaka used for histologic analysis (n = 6 per group). Bars denote standard deviation. Differences between groups were significant by Student's *t* -test (**, *P*<0.01).

The study design involved the comparison of individuals from three age groups: young adults, mature adults, and adults that had survived beyond the mean or median lifespan. To determine what ages were appropriate for this analysis, we performed a small-scale pilot study in which a cohort of 27 individual medaka fish was observed for 29 months post-hatching until 100% mortality was attained. A Kaplan-Meier survival analysis is shown in Fig. 1A. There was about 20% mortality at or near the time of hatching, with minimal mortality for the next 12 months. Mortality rate then increased with age, with the population going into steep decline after about 20 months. Mean and median lifespan and associated confidence intervals are shown in Table 1. Based on these results, we chose to use 6 month old, 16 month old, and 24 month old fish to represent the three age groups.

**Table 1:** Mean and Median Survival Time^a^.

Mean			Median		
Estimate	Std. error	95% C.I.^b^	Estimate	Std. error	95% C.I.
16.8	1.9	13.1–20.5	21.9	2.2	17.5–26.3

a Abbreviations are: Std. error, Standard error; C.I., Confidence interval.

Fig. 1B shows the gross appearance of representative individuals form each group. The fish grow throughout this period with little external signs of aging except for mild spinal curvature in some individuals in the 24 month-old group. Consistent with a prior report [20] this spinal curvature became more severe in the very oldest fish in the population, which also lost coloration (data not shown). To quantify the growth pattern, we determined the wet weight for six male individuals in each age group. Mean weight increased significantly at each time interval (P<0.01) (Fig. 1C), indicating continuous growth into old age.

### Age-associated changes in the liver

Age-associated changes have been widely reported in fish liver. Studies using various species have reported vacuolization, fibrosis, appearance of spindle-like cells with pyknotic nuclei, accumulation of lipofuscin, protein oxidation, and malignant transformation ([4], [28], reviewed in [1]).

**Figure 2:**
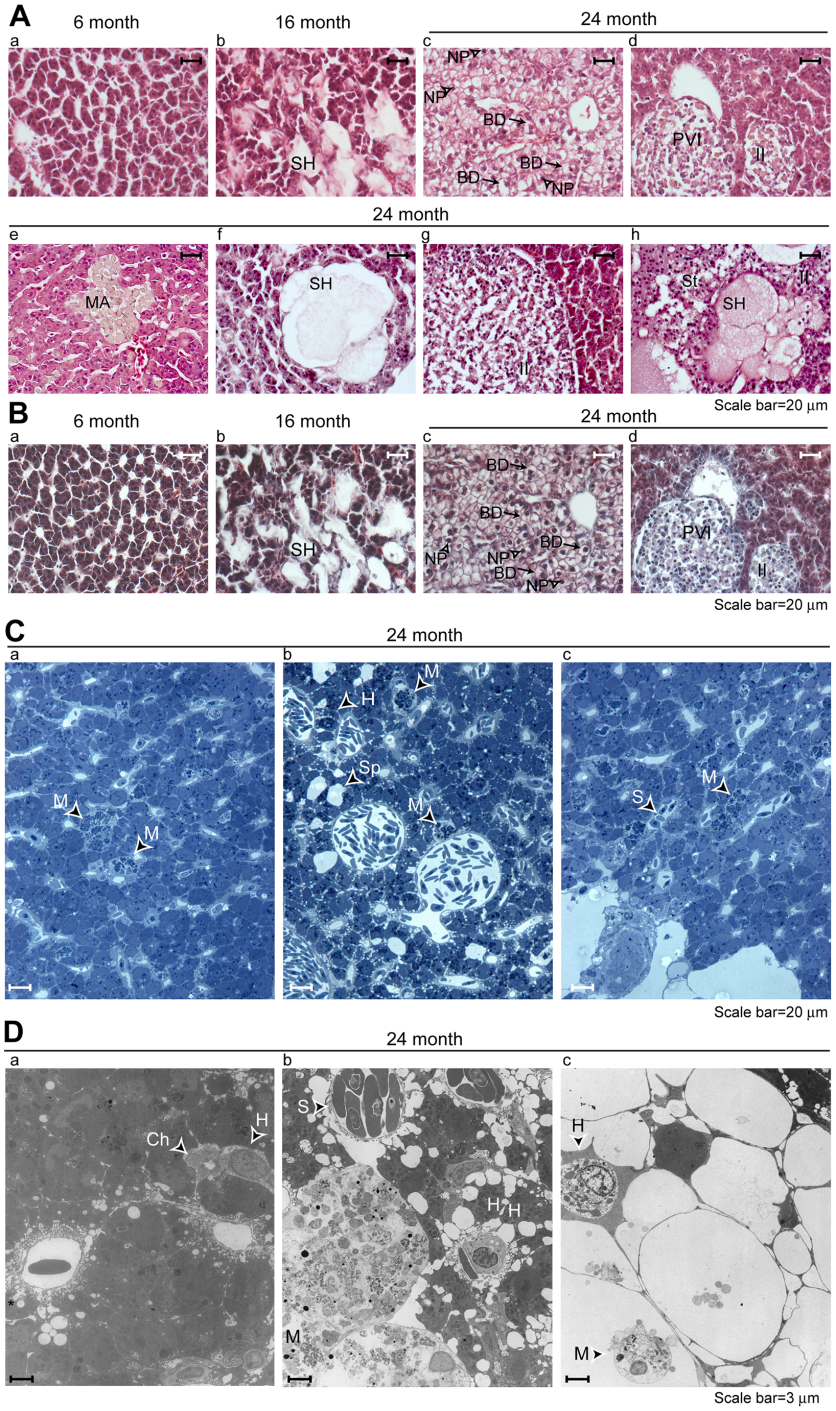
Age-associated morphological changes in liver. (**A-B**). Paraffin-embedded sections were prepared and stained as described in Materials and Methods. (**A**) Hematoxylin and eosin staining. (a-h) Individual sections representing pathology observed in different age groups. Lettering and symbols denote spongiosis hepatis (SH), ballooning degeneration (BD), nuclear pyknosis (NP), perivascular inflammation (PVI), inflammatory infiltrate (II), macrophage aggregate (MA), and steatosis (St). (**B**) Gomori's Trichrome stain. Panels (a) through (d) correspond to serial sections of corresponding specimens in (**A**). (**C**) Toluidine blue-stained semithin sections from 24-month old individuals. Lettering denotes macrophages (M), necrotic hepatocyte (H), space once occupied by hepatocyte (Sp), and congested sinusoid (S). (**D**) Transmission electron micrographs of liver sections from same fish. Lettering and symbols denote vacuoles communicating with space of Disse (*), bile cholangiole (Ch), macrophage (M), necrotic or shrunken hepatocytes (H), and congested sinusoid (S).

To evaluate age-associated morphological alterations in the medaka, we prepared paraffin-embedded histologic sections from nine individuals in each age group. Normal morphology, defined by a regular pattern of hepatocytes and sinusoidal spaces with an absence of vascular congestion, inflammatory infiltration, and cytoplasmic vacuolization, was seen in all of the 6-month old fish (Table 2). One of the 16-month old fish showed spongiosis hepatis, steatosis, macrophage aggregates and inflammatory infiltration. Four other 16-month old fish showed just one of these changes (either spongiosis hepatis or steatosis, but not both). The 24-month old fish showed widespread and severe pathology, including pyknotic nuclei in 8/9 individuals (*P*<0.01 vs. 16 month group), spongiosis hepatis in 9/9 individuals (*P* = 0.03 vs 16 month), and macrophage aggregates in 8/9 individuals (*P*<0.01 vs. 16 month). Some of the individuals showed steatosis, ballooning degeneration (characterized by enlargement, whispy cytoplasm, and central, pyknotic nuclei), inflammatory infiltrates, and perivascular inflammation. Representative micrographs (Fig. 2) illustrate areas with pathological changes (see Figure Legend for key to labeling). Although we did not attempt to quantify the changes, the micrographs of the 24-month-old specimens indicate many areas in which pathological changes predominated, and in some instances, little normal morphology remained. Serial sections of the same specimens were stained with Gomori's Trichrome Stain (Fig. 2B). Characteristic blue/black nuclei and variable, red to blue cytoplasm were seen, with no evidence of blue fibrotic lesions.

**Table 2:** Summary of Liver Pathology^a^.

Fish	NP	SH	BD	ST	II	MA	PVI
24 month	**+**	**+**	**+**	**+**	**-**	**+**	**-**
	**+**	**+**	**-**	**+**	**+**	**+**	**-**
	**+**	**+**	**-**	**+**	**+**	**+**	**-**
	**+**	**+**	**+**	**+**	**+**	**-**	**-**
	**+**	**+**	**-**	**+**	**+**	**+**	**+**
	**+**	**+**	**-**	**-**	**-**	**+**	**-**
	**+**	**+**	**-**	**+**	**-**	**+**	**-**
	**-**	**+**	**-**	**-**	**-**	**+**	**-**
	**+**	**+**	**-**	**+**	**-**	**+**	**-**
16 month	**-**	**+**	**-**	**+**	**+**	**+**	**-**
	**-**	**+**	**-**	**-**	**-**	**-**	**-**
	**-**	**-**	**-**	**-**	**-**	**-**	**-**
	**-**	**-**	**-**	**-**	**-**	**-**	**-**
	**-**	**-**	**-**	**-**	**-**	**-**	**-**
	**-**	**-**	**-**	**+**	**-**	**-**	**-**
	**-**	**+**	**-**	**-**	**-**	**-**	**-**
	**-**	**+**	**-**	**-**	**-**	**-**	**-**
	**-**	**-**	**-**	**-**	**-**	**-**	**-**
6 month	**-**	**-**	**-**	**-**	**-**	**-**	**-**
	**-**	**-**	**-**	**-**	**-**	**-**	**-**
	**-**	**-**	**-**	**-**	**-**	**-**	**-**
	**-**	**-**	**-**	**-**	**-**	**-**	**-**
	**-**	**-**	**-**	**-**	**-**	**-**	**-**
	**-**	**-**	**-**	**-**	**-**	**-**	**-**
	**-**	**-**	**-**	**-**	**-**	**-**	**-**
	**-**	**-**	**-**	**-**	**-**	**-**	**-**
	**-**	**-**	**-**	**-**	**-**	**-**	**-**

a Abbreviations are as follows: SH, spongiosis hepatis; MA, macrophage aggregrates; ST, steatosis; BD, ballooning degeneration; NP, nuclear pyknosis; II, inflammatory infiltration (lymphocytes, plasma cells, macrophages); PVI, perivascular inflammation.

We performed a complementary analysis of liver ultrastructure in specimens from 24-month old individuals. Samples were fixed and embedded in resin as described in Materials and Methods. Toluidine-blue stained semithin sections are shown in Fig. 2C. Panel 2C(a) shows less-altered hepatic parenchyma, characterized by small foci where hepatocytes appear to have been replaced by macrophages (M). Panel 2C(b) illustrates what appear to be different stages of the degenerative process, including one necrotic hepatocyte with no inflammatory response (H), two microinflammatory foci where hepatocytes appear to have been replaced by macrophages (M), and five open areas with some low density protein or other material in the space once occupied by the hepatocyte (Sp). This field also shows prominent congested venules containing nucleated erythrocytes. Panel 2C(c) shows a more-altered area of hepatic parenchyma with inflammatory foci consisting of macrophages (M), a congested sinusoid (S) containing nucleated erythrocytes, and a prominent area of spongiosis hepatis at lower left. Some hepatocytes appear necrotic, others have a less-densely stained area in the central portion of the cell (likely glycogen).

Transmission electron microscopy was performed using ultrathin sections (Fig. 2D). Panel 2D(a) shows less-altered parenchyma. One cell shows clear vacuoles (not lipid) at its basal face and communicating with space of Disse (asterisk). Hepatocytes contain numerous phagolysomes and residual bodies. A bile cholangiole (Ch) is adjacent to a necrotic hepatocyte (H). Panel 2D(b) shows more-altered hepatic parenchyma. At lower left corner, hepatocytes have been replaced by large macrophages containing phagocytic debris (M). Parenchyma at top and right hand portion of field shows shrunken hepatocytes (H) devoid of lipid but revealing clear vacuoles at their peripheries. The sinusoids (S) at top of field are congested with nucleated erythrocytes. Panel 2D(c) shows strikingly altered parenchyma characterized by spongiosis hepatis. Except for the single necrotic hepatocyte (H) at left of field, spaces where hepatocytes once resided are now filled with very low electron density transudate and necrotic debris. At lower left of field, necrotic debris has been phagocytosed by the macrophage (M). The hepatic skeleton remains, composed of stellate cells with highly attenuated cytoplasm.

**Figure 3:**
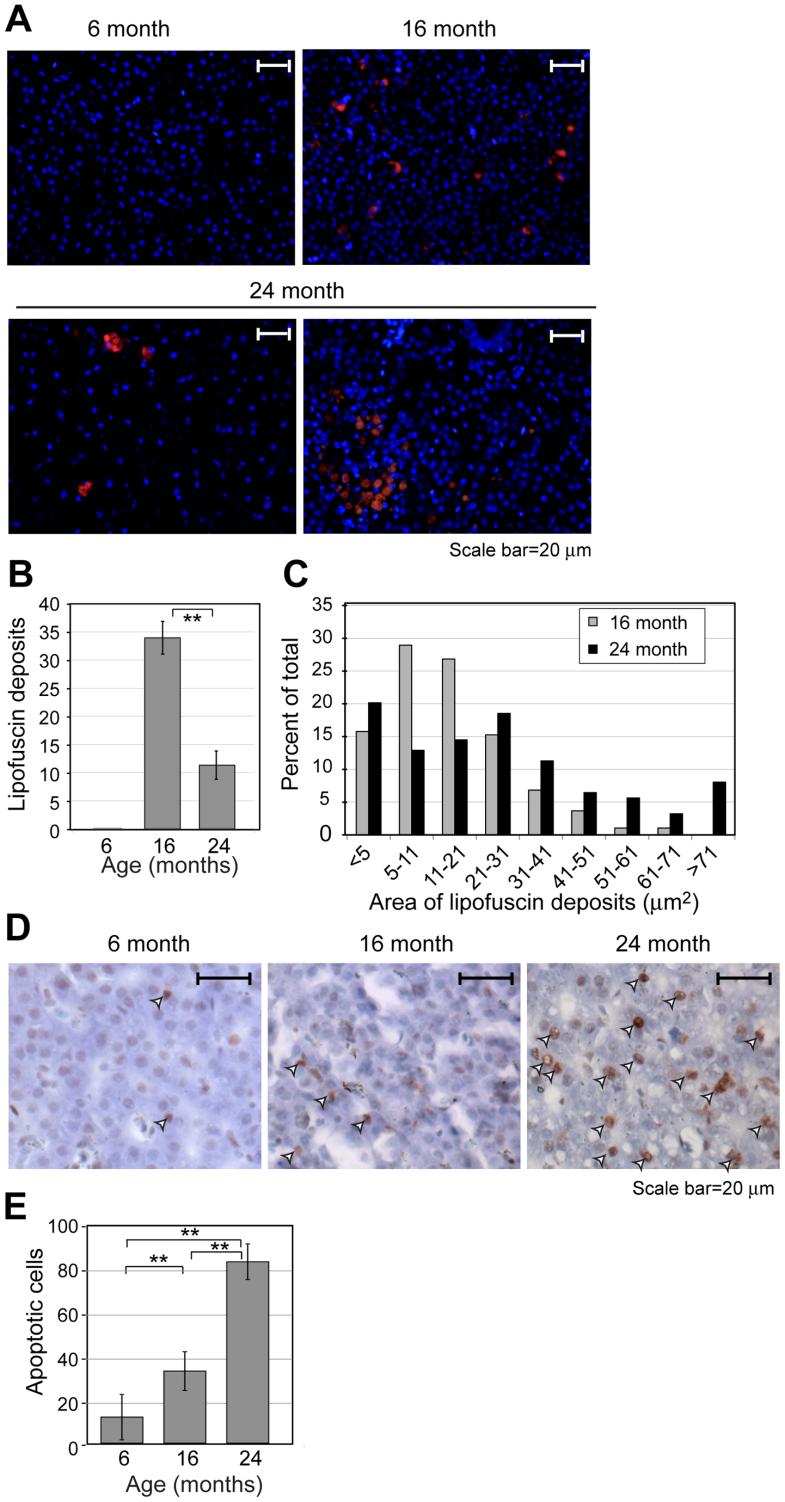
Quantifiable biomarkers in aging liver. (**A**) Detection of lipofuscin by autofluorescence (red channel). Nuclei were counterstained with DAPI (blue channel). (**B**) Quantification of lipofuscin deposits per field. Number of deposits per field was scored manually and areas were determined as described in Materials and Methods. Error bars denote standard deviation. Significance was determined based on Student's *t*-test (**, *P*<0.01). Each field is 2.13×10^3^ µm^2^. (**C**) Area of lipofuscin deposits. (**D**) Detection of apoptotic cells based on TUNEL staining (see Materials and Methods) and condensed nuclear morphology. Nuclei were counterstained with methyl green. Positive cells are denoted by arrowheads. (**E**) Quantification of apoptotic cells per field. Each field is 3.29×10^4^ µm^2^. Error bars and significance as in panel B (**, *P*<0.01).

To identify quantifiable biomarkers in liver, we investigated the presence of autofluorescent lipofuscin deposits (Fig. 3A). Experiments were performed using paraffin-embedded liver sections. Nuclei were counterstained with DAPI. Prominent red autofluorescent granules were seen in the 16 and 24 month-old specimens, but not in the 6 month-old specimens. Quantification showed that the number of lipofuscin deposits was highest in the 16 month-old fish, with a significant decline in the 24 month-old fish (Fig. 3B). As the total number of deposits declined, the average size increased (Fig. 3C), suggestive of consolidation of smaller deposits into larger aggregates.

We also investigated the presence of apoptotic cells based on TUNEL staining and nuclear morphology. Apoptotic cells (arrowheads) were sparse in the specimens from 6-month old fish, somewhat more abundant in those from 16-month old fish, and frequent in those from 24-month old fish (Fig. 3D). Quantification showed that the number of apoptotic cells increased significantly at each timepoint (Fig. 3E).

### Age-associated changes in skin and muscle

Aging has profound effects on both skin and skeletal muscle in many organisms. Skin and skeletal muscle have previously been examined in aging zebrafish [6]. SA β-gal, a widely used biomarker of vertebrate aging [10], [29], was prominently visible in the skin of aged zebrafish. Somewhat mixed results were reported for zebrafish skeletal muscle. Analysis of muscle extracts indicated accumulation of oxidized protein. Lipofuscin deposits were not detected, however, and proliferative capacity, as measured by BrdU incorporation was maintained [6].

**Figure 4:**
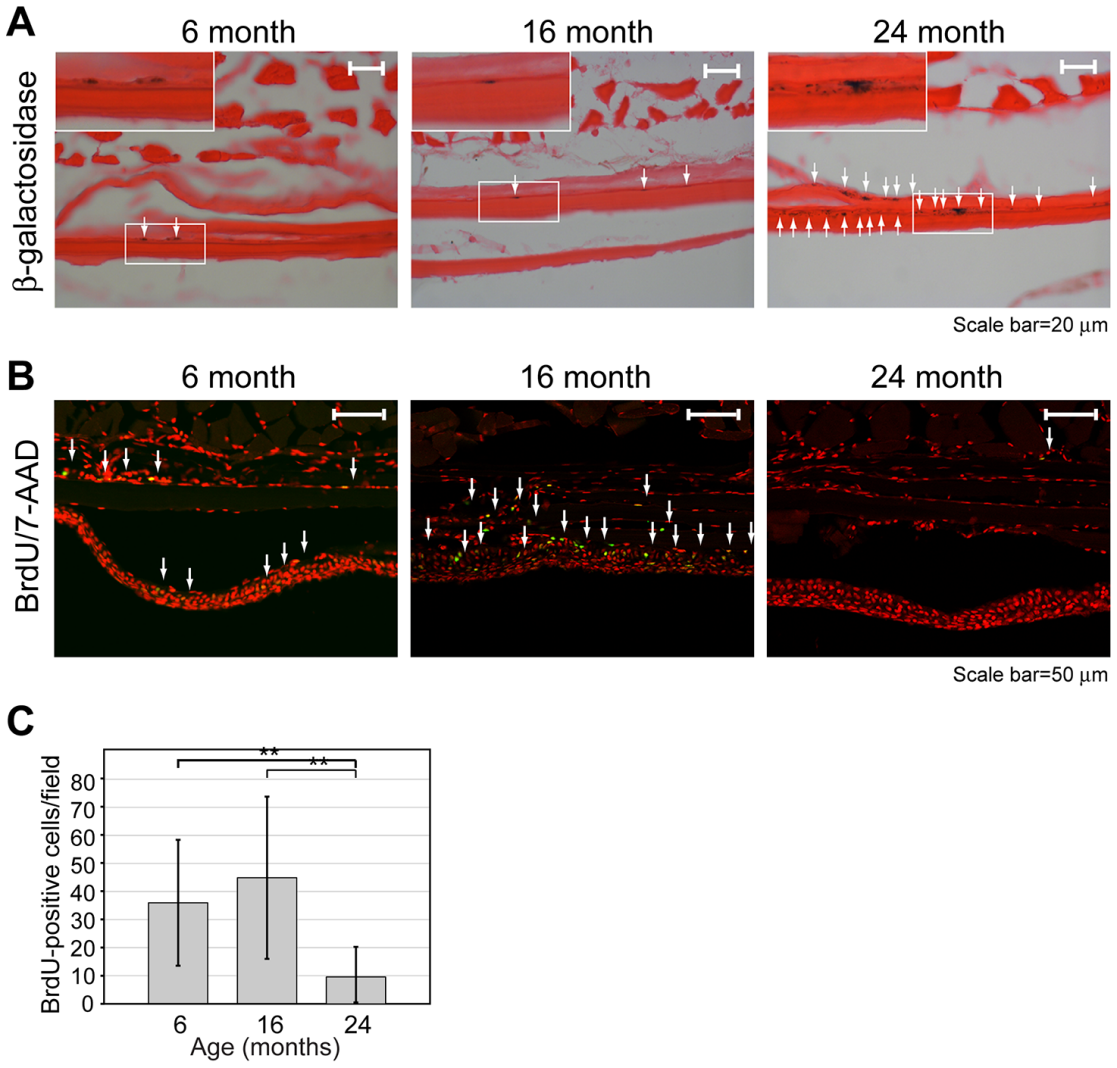
BrdU incorporation and senescence-associated β-galactosidase activity in skin and skeletal muscle. Each panel is a transverse section showing skeletal muscle (at top) and skin (at bottom). Cryosections were prepared and stained as described in Materials and Methods. (**A**) Senescence-associated β-galactosidase detected by histochemical analysis using X-gal substrate (blue staining). Positive areas are denoted by white arrows. Tissue was counterstained with eosin. Insets show higher magnification views of boxed regions. (**B**) BrdU incorporation detected by immunofluorescence (green channel). Positive cells are denoted by white arrows. Nuclei were counterstained with 7-amino actinomycin D (7-AAD) (red channel). (**C**) BrdU-positive cells per field were quantified as described in Materials and Methods. Error bars denote standard deviation. Significance was determined based on Student's *t*-test (**, *P*<0.01). Each field is 2.26×10^5^ µm^2^.

To investigate age-associated changes in skin and skeletal muscle of medaka, we prepared frozen sections from fish that had been prelabeled for 7 days with BrdU. Staining results for SA β-galactosidase are shown in Fig. 4A. Positive areas (arrows) were most numerous in the 24 month-old samples, with only sparse labeling of 6 and 16 month-old samples. Sections from the same individuals were also stained with anti-BrdU antibody (Fig. 4B). Brightly labeled cells (arrows) in the basal layer of the dermis were present in 6 month-old fish, prominent in 16 month-old fish, and apparently absent at 24 months. Quantification of the same data is shown in Fig. 4C. The number of BrdU-positive cells declined significantly in the oldest individuals. It is notable that BrdU staining was seen principally in the basal layer of the dermis, that is, the same layer where SA β-gal was seen in samples from older individuals. Together, data are indicative of a reciprocal relationship between SA β-gal staining and reduced proliferative capacity as measured by BrdU labeling.

Age-associated changes were not apparent in skeletal muscle, which was present in the same sections. In this tissue, there was only sparse BrdU labeling (1–2 cells per microscope field, Fig. 4B and data not shown), which did not change with age. The apparent absence of age-related degeneration in skeletal muscle is consistent with the continuous increase in body size and mass seen in Figs. 1B and 1C.

### Age-associated changes in the heart

**Figure 5:**
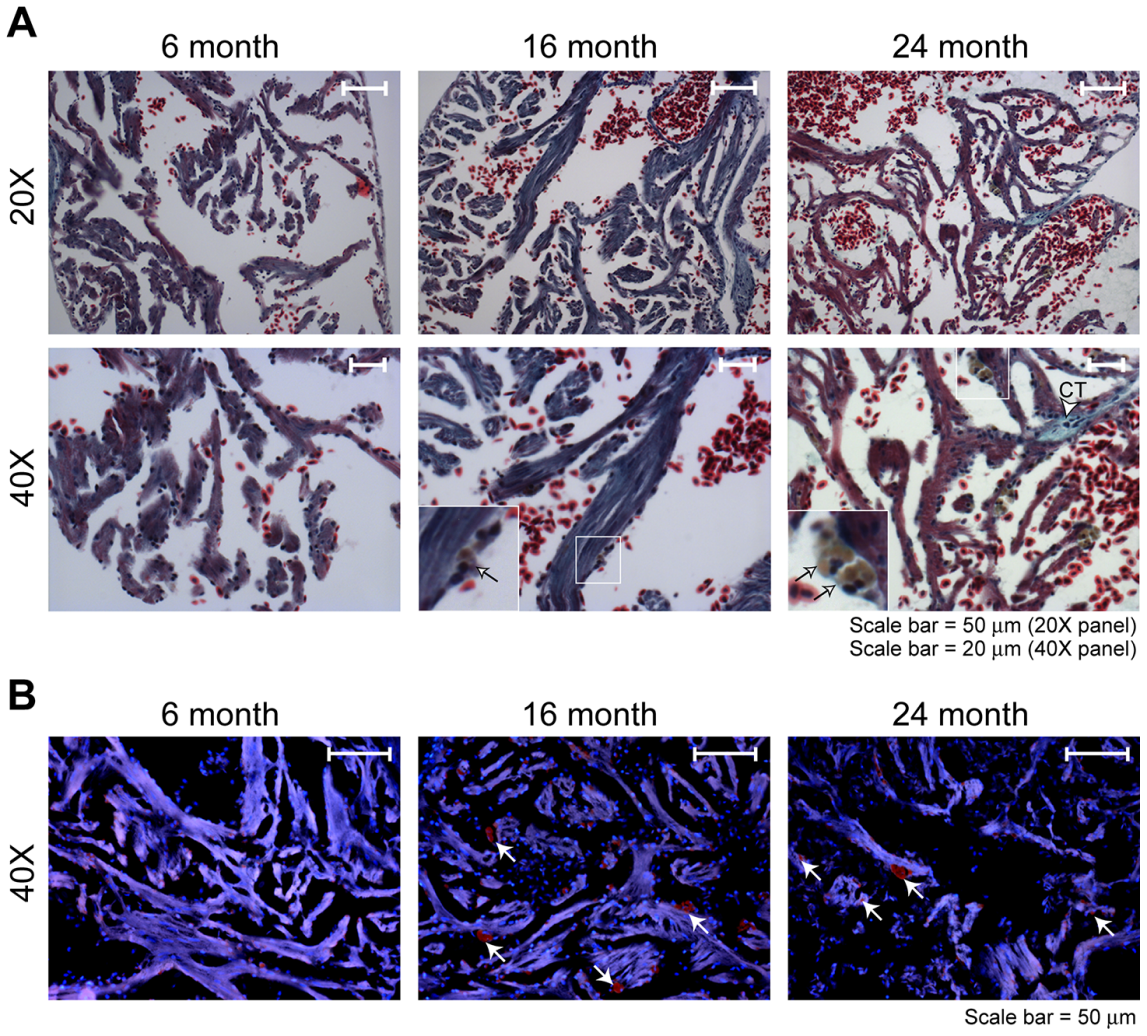
Histology and lipofuscin accumulation in heart. Paraffin-embedded sections were prepared and stained as described in Materials and Methods. (**A**) Gomori's Trichrome staining was performed as described in Materials and Methods. Arrows in 40X images indicate lipofuscin granules. Open arrowhead (40X image of 24 month-old fish) indicates blue-stained connective tissue (CT). Insets show enlargements of boxed regions (**B**) Detection of lipofuscin by autofluorescence (red channel, deposits indicated by white arrows). Nuclei were counterstained with DAPI (blue channel).

There has been a report of age-associated changes in the heart of the aging guppy fish [30]. To investigate age-associated changes in the medaka heart, we used Gomori's Trichrome stain, which simultaneously indicates nuclei (dark blue/black), erythrocytes and muscle fibers (shades of red/blue), and connective tissue (blue/green) [31] (Fig. 5A). The hearts of the 6 month-old group appeared normal with no evidence of fibrosis or lipofuscin deposits. There were occasional brown deposits visible in the heart of 16 month-old fish (arrows, inset), which we interpreted as lipofuscin. Hearts of the 24 month-old fish showed many, larger brown lipofuscin deposits (arrows) and blue-stained connective tissue (open arrowhead).

The accumulation of lipofuscin was confirmed by autofluorescence. Prominent red autofluorescent granules were seen in the 16 and 24 month-old specimens (Fig. 5B, arrows). By contrast, few or no red autofluorescent granules are seen in the 6 month-old specimen. Together, results indicate a difference between cardiac muscle, where age-associated degenerative changes were evident, and skeletal muscle, where no age-associated changes were seen (Fig. 4).

### Age-associated changes in the eye

**Figure 6:**
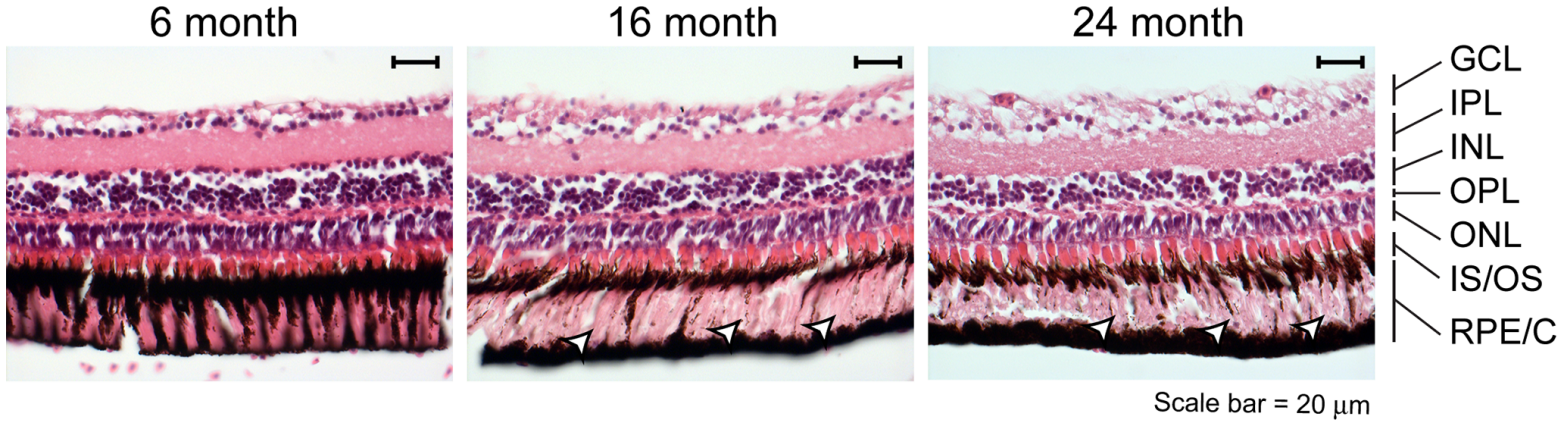
Histology of retina. Each panel is a transverse section of a similar region of the retina. Paraffin embedded sections were stained with hematoxylin and eosin as described in Materials and Methods. Cell layers are indicated at right: GCL, ganglion cell layer; IPL, inner plexiform layer; INL, inner nuclear layer; OPL, outer plexiform layer; ONL, outer nuclear layer; IS/OS– inner segment/outer segment of the photoreceptor cells; RPE/C, retinal pigment epithelium/cells. Arrowheads in center and right panel indicated regions where pigmented cells have been lost.

Age-associated degenerative changes in the eye occur prominently in many vertebrates, including zebrafish. To investigate whether such changes occur in the medaka, we examined the histology of the retina in fish from different age groups (Fig. 6). We observed consistent changes in the retinal pigment epithelium/cell layer, with reduction or loss in the number of strongly pigmented cells seen in the 16 month and 24 month-old specimens (arrowheads). Age-associated changes in the other retinal layers were not consistently seen, however. In zebrafish, there has been a report of age-associated accumulation of lipofuscin, and the occurrence of drusen-like lesions, similar to those seen in age-related macular degeneration in humans [4]. We did not observe these phenomena in our medaka cohort (data not shown). We also did not observe age-related increases in lens opacity, which would be indicative of cataractogenesis (data not shown).

## Discussion

In the present study, we characterized biomarkers of normal aging in a population of medaka fish that was maintained under laboratory conditions. We define aging in terms of the survival curve, which steeply declined after about 20 months. Among the organs examined, the earliest and most extensive signs of aging were seen in the liver. Morphologic examination using light and transmission electron microscopy showed many changes at the level of individual hepatocytes. Death of these cells appears to trigger formation of small inflammatory foci, with individual macrophages replacing hepatocytes. Aged liver was also characterized by congestion of the blood vessels. In some specimens, larger areas of inflammation with infiltration of lymphocytes were seen. Spongiosis hepatis (also called cystic degeneration), which consists of cyst-like, multilocular formations, filled in some cases with acidophilic material, may represent an end stage in this degenerative process. Quantitative histologic analysis revealed a significant increase in the number and size of autofluorescent lipofuscin granules, and a rise in the proportion of TUNEL-positive apoptotic cells.

We did not observe neoplasia in any of the liver sections that were examined, consistent with the reported low incidence of spontaneous liver neoplasia in medaka, especially among males [32], [33]. It has been suggested that spongiosis hepatis, which was observed, may be a preneoplastic lesion, although this is controversial [34], [35]. Spongiosis hepatis has been extensively studied in aging rats (reviewed in [34]) and has been reported in several previous studies of medaka and other teleosts [21], [36], [37], although it apparently does not occur in humans or mice. Carcinogen exposure can increase the incidence of spongiosis hepatis, and the lesion is sometimes found in association with hepatic neoplasia. However, direct evidence that it is a precursor to neoplasia, particularly in teleosts, is lacking.

Age-associated changes were also seen in other tissues, although they were not as prominent as in liver. There was an age-associated decline in the number of BrdU-labeled proliferative cells and an increase in SA β-gal in the basal layer of the dermis. Age-associated changes in the heart were noted, including accumulation of lipofuscin granules, loss of muscle fibers, and presence of fibrotic tissue. The retinal epithelium showed an apparent loss of pigmented cells. Age-associated changes were not observed in skeletal muscle, ocular lens, or brain (data not shown). We cannot rule out the possibility that age-associated changes in these or other tissue might be seen if a larger number of animals were observed.

Zebrafish have been reported to show some of the same age-associated tissue alterations as reported here for the medaka, including pathologic changes in liver and accumulation of SA β-gal in the dermis [4], [6]. There are also some differences, however. Accumulation of SA β-gal is prominently visible in whole-mount preparations of zebrafish and has been used as the basis of a genetic screen [10]. We were not successful in applying this method in medaka, as high levels of background were present in all specimens with no apparent differences between age groups. Zebrafish reportedly exhibit elevated levels of oxidized protein in aging skeletal muscle [6], which we were unable to detect in medaka (data not shown). Zebrafish have also been reported to undergo age-dependent changes in the abundance and aggregation of lens crystallins [38]. We did not examine lens proteins in the medaka, although gross changes in the lens were not apparent. We also did not observe morphologic changes in the retina to the same extent as has been reported in very old zebrafish [6], although we cannot exclude the possibility that these might have been observed if we had examined the very oldest medaka.

Several general theories have been proposed to explain the root causes of aging, including cumulative effects of oxidative stress. It seems likely that oxidative stress contributes to aging in the medaka, based on the presence of lipofuscin granules, composed mostly of peroxidized lipids, in both the liver and heart. Exhaustion of stem/progenitor cells is also a possible contributor to aging in the medaka, consistent with loss of BrdU-labeled stem/progenitor cells in the basal layer of the dermis. Whether telomere erosion contributes to exhaustion of stem/progenitor cells, as has been posited in other organisms, is unknown. In contrast to mammalian systems, telomerase is present in somatic adult tissue of the medaka [18], [19], [20], although age-associated telomere shortening has also been reported in medaka [20].

The lifespan of poikilothermic organisms is inversely correlated with temperature [39], [40], [41]. This may, in part, reflect the dependence of oxidative metabolism and resultant oxidative stress on environmental temperature, although other factors also play a role ([42], reviewed in [41]). Medaka in the present study were maintained under standard laboratory conditions at constant temperature (27±1°C) and with a uniform light:dark cycle; they do not undergo a period of winter dormancy as they would in their natural environment. Although the experimental design is somewhat different, it appears that the median lifespan observed here is somewhat shorter than in medaka studies performed by Egami and coworkers in the 1960s and 1970s [17], which were conducted in an outdoor environment. Although maintenance of the medaka under controlled laboratory conditions, with the shorter lifespan, is conducive to experimental studies, it is worth noting that the specifics of the aging process (including the organ systems that are most affected) may be different than in the wild. Similar considerations apply to other model organisms used in aging research.

One rationale for the use of small laboratory fish as models for aging is the well-developed genetics. Mutant lines of medaka have been developed that affect *BLM*, which is associated with progeria in humans, *PARK2*, which is associated with hereditary Parkinson's disease, *SIRT1*, which is the homolog of a gene that affects aging in budding yeast, and *TP53*, which is associated with Li-Fraumeni syndrome and frequently undergoes somatic mutation in cancer [43]. Clearly, it will be of interest to investigate the effect of mutations in these and other genes on the endpoints reported here. Medaka has also been widely used as a model for toxicology research (for example, [21], [22]). There has been at least one study linking genotoxic insult to accelerated aging in the medaka [16]. In this work, report that a 1 Gy dose of radiation (about 5% of the LD_50_ [44], [45]), delivered to embryos at 8d post-fertilization, increased mortality rates at times long after exposure. Clearly, it will be of interest to investigate effects of radiation and other genotoxic exposures on quantitative biomarkers of aging.

## Materials and Methods

### Fish

CAB wild-type Japanese medaka fish (*Oryzias latipes*) were maintained with a 12 h:12 h light: dark photoperiod at 27±1°C in a recirculating habitat system. Fish were maintained in conditioned water with quality parameters as follows: pH, 7.5–8.3; conductivity, 500–560 µS; alkalinity, 80–100 mg/L as CaCO_3_; hardness, 100–120 mg/L as CaCO_3_; and dissolved oxygen, 5–7 mg/L. Fish were fed freely until they reached satiation twice daily, once with brine shrimp plus flake food in the morning and once with flake food in the afternoon. Protocols were approved by the Medical College of Georgia Institutional Animal Care and Use Committee (protocol number BR09-10-259), which specifically approved this study.

### Statistical methods

Statistical analysis was performed using the SPSS package (IBM Corp., Chicago, IL).

### Experimental design and tissue processing

The lifespan study was based on a group of 27 viable embryos, which were allowed to hatch and followed until 100% mortality was reached at 29 months. The remaining studies were performed using individuals that were selected at random from separate, age-grouped populations maintained in the same habitat. To minimize subjectivity, a person who was not otherwise involved in the study performed this selection. Some individuals were subjected to a BrdU labeling procedure (as described in a later section of Materials and Methods) and others were euthanized and tissues collected immediately. No differences in histology of paraffin-embedded tissues were apparent among individuals that were or were not subjected to the BrdU labeling procedure, and thus histologic data from all individuals within each age group was pooled in the analysis.

After fixation, liver, heart, and eye tissue were collected and paraffin-embedded. Sections (5 µm) were prepared, then dewaxed prior to staining. Staining was performed using hematoxylin and eosin or a Gomori's Trichrome Stain kit (Polysciences, Inc., Warrington, PA) as indicated in the Figure Legends. For Gomori's Trichrome staining, slides were incubated in Bouin's fixative overnight at room temperature. Samples were washed to remove all yellow color and stained with Wiegert's Iron Hematoxylin for 10 min at room temperature. Samples were again washed and stained with Gomori's Trichrome Stain for 15 min at room temperature. Samples were then placed in 0.5% hydrochloric acid for 2 min to differentiate the stain. Samples were dehydrated through graded ethanols and cleared in xylene. Liver pathology was interpreted according to [21].

For analysis of hepatic ultrastructure, a portion of the liver was reserved, prior to paraffin embedding, and transferred to PBS containing 15% sucrose, and kept at 4°C until processing.

For evaluation of hepatic ultrastructure, 0.5 mm thick slices were made from the periphery of each liver, minced into 0.5–1 mm rectangles, and placed in fresh fixative. With initiation of processing, rectangles were rinsed twice in 0.1 M sodium phosphate buffer (pH 7.2–7.4) and post-fixed in the same buffer containing 1% osmium tetroxide for 1 h at room temperature. The resultant secondarily fixed tissue pieces were rinsed twice in distilled water, dehydrated in graded ethanol, rinsed twice in 100% acetone, placed in a 1∶1 mixture of Spurr's resin and acetone for 30 min, and then transferred to 100% Spurr's resin for 1 h. Tissue was transferred to fresh 100% Spurr's resin for 1 h, placed in molds and allowed to polymerize at 70°C overnight. Semithin sections (500 nm thick) were cut with glass knives and stained with 1% Toluidine Blue O in 1% sodium borate. Once liver was verified as present in semithin sections, ultrathin sections (70–90 nm thick) were cut with a diamond knife, stained with methanolic urany acetate and lead citrate, and examined using a FEI/Philips EM 208S transmission electron microscope at 80 kV accelerating voltage. Transmission electron microscopy, processing, analysis, and imaging were performed at the Laboratory for Advanced Electron and Light Optical Methods, College of Veterinary Medicine, North Carolina State University, Raleigh, NC.

### Lipofuscin detection

Paraffin-embedded sections were dewaxed, rehydrated, stained with 0.6 µM 4′,6-diamidino-2-phenylindole (DAPI) and mounted with Antifade media (Invitrogen). Lipofuscin autofluorescence was detected at 585 nm and DAPI fluorescence was detected at 405 nm. Nine fish per age group were examined and representative images are shown. For quantification of lipofuscin deposits in liver, 10 randomly selected, nonoverlapping fields per individual were scored manually (each field is 2.13×10^3^ µm^2^). Areas were calculated using the ZEN 2009 LE software package (Carl Zeiss Microimaging, Inc, Thornwood, NY).

### TUNEL assay

Paraffin-embedded sections were dewaxed and rehydrated. Samples were then pretreated by heating to boiling for 10 minutes in 0.01 M citrate buffer pH 6.0. Heating was performed using a 1000 W microwave oven at 100% power for 1 min to bring the samples to boiling and 30% power for 9 min to maintain temperature. Endogenous peroxidase was quenched and TdT labeling was performed using a ApopTag peroxidase *in situ* apoptosis detection kit (Millipore, Billerica, MA). Slides were counterstained with 0.5% methyl green, dehydrated, and mounted. Nine fish per age group were examined. 10 randomly selected fields (3.29×10^4^ µm^2^ each) per individual were scored manually. Cells were scored as apoptotic only if both peroxidase staining and condensed nuclear morphology were seen.

### BrdU incorporation assay

Three individuals from each age group were maintained in foil-covered beakers (300 ml of conditioned water in a 600 ml beaker) with 80 mg/L BrdU (Sigma-Aldrich, St. Louis MO) for 7 days. They were fed once with brine shrimp on day 4, with a change of water 2 hours after feeding. Three control individuals in each age group were maintained identically except in the absence of BrdU. Fish were euthanized on day 8 of incubation, fixed in 2% paraformaldehyde, 0.05% glutaraldehyde, 80% Histochoice (Electron Microscopy Sciences, Hatfield, PA), 1% sucrose and 1% CaCl_2_ at 4°C overnight and incubated with gum sucrose (0.88 M sucrose containing 1% gum acacia) at 4°C for a further 48 hours [46]. The trunk, containing skeletal muscle and skin, was embedded in Tissue-Tek Optimal Cutting Temperature (OCT) compound (Fisher Scientific, Pittsburgh, PA). OCT embedded tissue was kept at −80°C before sectioning.

For evaluation of BrdU uptake in skeletal muscle and skin, 8 µm transverse sections were prepared from OCT-embedded tissues. Sections were placed on Superfrost Plus slides (Fisher Scientific, Pittsburgh, PA) and prepared for staining as described [6]. Samples were then incubated overnight at 4°C with anti-BrdU mouse monoclonal antibody PRB-1 (Invitrogen, Carlsbad, CA), 1∶50 dilution in blocking buffer (PBS containing 1% Tween 20, 10% fetal calf serum, 1% DMSO). Negative control samples were incubated in blocking buffer without primary antibody. Slides were washed with PBS and incubated for 1 h at 37°C with Alexa Fluor 488-conjugated goat anti-mouse IgG1 (Invitrogen), 1∶200 dilution in blocking buffer containing 32 µM 7-amino actinomycin D. Slides were washed, mounted with Prolong Gold Antifade Reagent (Invitrogen) and stored in the dark at 4°C. Images were collected and BrdU-positive cells were scored manually. A minimum of 11 non-overlapping fields (2.26×10^5^ µm^2^ each) per age group were scored.

### Senescence-associated β-galactosidase assay

Cryosections were prepared and placed on slides as described in the preceding section. Slides were stained as described [29]. Briefly, slides were stained overnight at 37°C with a solution of 1 mg/ml 5-bromo-4-chloro-3-indolyl-β-D-galactopyranoside (X-gal), 5 mM potassium ferricyanide, 40 mM citric acid-sodium phosphate pH 6.0, 2 mM MgCl_2_, 150 mM NaCl. Slides were washed three times with PBS and once with water, counterstained with eosin, dehydrated, and mounted. Four fish per age group were examined and representative images are shown.
